# Incidence and predictors of loss to follow-up among HIV infected adults after initiation of first line anti-retroviral therapy at University of Gondar comprehensive specialized Hospital Northwest Ethiopia, 2018: retrospective follow up study

**DOI:** 10.1186/s13104-019-4154-y

**Published:** 2019-02-28

**Authors:** Nebiyu Mekonnen, Mohamed Abdulkadir, Eleyias Shumetie, Adhanom Gebreegziabher Baraki, Melaku Kindie Yenit

**Affiliations:** 1 School of Medicine, College of Medicine and Health Sciences, University of Gondar, Gondar, Ethiopia; 2 Department of Epidemiology and Biostatistics, Institute of Public Health, College of Medicine and Health Sciences, University of Gondar, Gondar, Ethiopia

**Keywords:** HIV, Antiretroviral therapy, Loss to follow up, Northwest Ethiopia

## Abstract

**Objectives:**

The aim of this study was to estimate the incidence of lost to follow up from anti-retroviral therapy (ART) care and identify the associated factors among human immunodeficiency virus (HIV) infected patients after first-line ART initiation at University of Gondar comprehensive specialized hospital, Northwest Ethiopia between January 2012 and January 2018.

**Results:**

The overall incidence rate of lost to follow up was 12.26 per 100 person years (95% CI (10.61–14.18)). Being underweight (< 18.5 kg/m^2^) (AHR, 1.52, 95% CI 1.01–2.28), jobless (AHR, 2.22, 95% CI 1.2–4.11), substance abuser (AHR, 1.84 95% CI 1.19–2.86), having sub-optimal adherence (fair/poor) (AHR 6.33, 95% CI (3.90–10.26)), not receiving isoniazid prophylaxis (AHR 2.47, 95% CI (1.36–4.48)), ambulatory functional status (AHR 1.94, 95% CI (1.23–3.06)), having opportunistic infections (AHR, 1.74 95% CI 1.11–2.72), having CD4 count 201–349 cells/µL (AHR 0.58, 95% CI (0.38–0.88)) were found to be significant predictors of lost to follow up from ART service.

## Introduction

Human immunodeficiency virus/acquired immunodeficiency syndrome (HIV/AIDS) is the greatest worldwide public health problem. Even though the disease has no cure the global scale-up of ART has contributed to full preventive and therapeutic benefits [1]. ART contributed to a 32% and 16% global decline in AIDS-related deaths and new HIV infections respectively between 2010 and 2016 [1, 2].

In 2015 World Health Organization (WHO) recommended “treat all policy” that all people living with HIV should start ART after confirmed HIV diagnosis and clinical assessment irrespective of WHO clinical stage or CD4 cell count [3]. Early initiation of ART provides maximal and durable viral load suppression, restore and preserve immune function, improve quality of life [4, 5], and prevent transmission [6].

According to the United States Agency for International Development (USAID) Fast-Track strategy 90% of people on ART should achieve sustained viral suppression with good adherence and retention to ART follow up [7]. But poor retention in care and lost to follow-up (LTFU) are great challenges in achieving this target [1]. LTFU was also highly associated with early death [8–11].

A systematic review conducted in sub-Saharan Africa in 2010 determined LTFU as the most common cause of attrition from HIV care (59%) followed by death (41%) and median attrition at 12, 24 and 36 months was 22.6%, 25% and 29.5% respectively. Retention was also decreased from 86.1% at 6 months to 64.6% at 36 months [12].

Lost to follow up from HIV care was positively associated with young age [9, 13, 14], male sex [14, 15] single marital status [13, 16], illiteracy [13, 16], long travel time to the clinic [14, 17], bedridden functional status [18, 19], presence of opportunistic infections (OIs) and opportunistic infection prophylaxis [20, 21] low CD4 count [9, 18, 22] less advanced WHO clinical stage [21].

The magnitude of LTFU and the factors contributing can vary from place to place. Early identification of the magnitude and factors is important to identify the vital intervention areas and improve the life of people living with HIV (PLHIV) via improved viral suppression. Even though there are few studies done Ethiopia, the rate of LTFU and its major predictors are not identified in the study area. So this study will fill this information gap.

## Main text

### Methods

A retrospective follow up study was conducted among HIV infected adults on first line ART at University of Gondar comprehensive specialized Hospital Northwest Ethiopia from January 2012 to January 2018. The Hospital is located in Gondar town, 727.22 km far from Addis Ababa, the capital city of Ethiopia. Gondar University comprehensive specialized hospitals began free ART service provision in March 2005, and since then 14,375 PLHIV were enrolled for care and treatment.

A total of 569 adult patient charts that is above 15 year old were reviewed after selecting them by simple random sampling from the ART register used in the ART clinic.

Trained nurses collected the data using a structured data extraction tool. The data were collected from patient charts. Data about the initiation time of therapy, the time when there was LTFU, socio-demographic, behavioral, clinical, therapeutic and immunologic factors were collected. LTFU was considered when a patient is not seen at the clinic for at least 90 days (3 months) after the last missed appointment but not transferred out from the facility to another facility or died. Time to LTFU was the time interval between the dates of ART initiation to the last missed appointment. Adherence was measured as follow; Good adherence: ≥ 95% adherence that is, missing only 1 out of 30 doses or missing 2 from the 60 doses. Fair adherence: 85–94% adherence that is, missing 2–4 doses out of 30 tabs or 4 to 9 tablets from 60 doses. Poor adherence: less than 85% adherence that is, missing ≥ 5 tablets out of 30 tabs or > 10 tabs from 60 tabs [6]. Functional status of participants was assessed based on WHO criteria as follows. Working: able to perform usual work inside or outside home. Ambulatory: able to perform activity of daily living-ADL, Not able to work. Bedridden: not able to perform ADL [23]. Substance abuse was measured as any history of harmful or hazardous use of psychoactive substances, including alcohol and illicit drugs. Censored are patients who died while on treatment, transferred out, or who are not lost from follow up till the end of the study period.

After collection data was cleansed, coded and entered into Epi Info version 7. Then it was exported to STATA 14 for further analysis. Person-year of follow up was calculated by using the time interval between the date of ART initiation and the date of LTFU or date of censoring. The Kaplan–Meier method was used to estimate rates of LTFU at specific time after ART initiation and Nelson–Aalen method was used to generate a cumulative hazard function. In the bivariable analysis with Cox proportional hazards regression model we selected independent predictors for multivariable Cox proportional hazards regression at “P” value less than 0.2. We used a stepwise backward selection procedure to identify socio-demographic and clinical independent predictors for LTFU at P < 0.05 adjusted hazard ratios (AHR) with 95% confidence interval was used as the measure of association.

### Results

#### Socio-demographic and behavioral characteristics

A total of 569 adult patients were followed for median follow-up time of 32 months (IQR = 40). Majority of the study participants 324 (60.1%) were females. Large proportion 227 (39.9%) of the participants were in the age category of 25–34 years. The majority 409 (71.9%) of participants were urban dwellers and most 514 (90.3%) were orthodox Christians. Of the total, 257 (45.2%) were married and 201 (35.3%) have attended primary education. The majority of participants 503 (88.4%) disclosed their HIV status at least to one individual (Table 1).

**Table 1:** Baseline socio-demographic characteristics of HIV patients on ART at University of Gondar compressive specialized Hospital ART clinic between January 2012 and January 2018

Characteristics	Category	Frequency (%)
Age in years	15–24	68 (12)
	25–34	227 (39.9)
	35–44	192 (33.7)
	> 45	82 (14.4)
Sex	Male	227 (39.9)
	Female	342 (60.1)
Marital status	Married	257 (45.2)
	Never married	83 (14.6)
	Divorced	159 (27.9)
	Widowed/separated	70 (12.3)
Religion	Orthodox	514 (90.3)
	Muslim	45 (7.9)
	Protestant	10 (1.8)
Residence	Urban	409 (71.9)
	Rural	160 (28.1)
Educational status	No education	154 (27.1)
	Primary	201 (35.3)
	Secondary	142 (25)
	Tertiary	72 (12.7)
Occupations	Government/NGOs employed	108 (19.0)
	Self employed	137 (24.1)
	Jobless	106 (18.6)
	Daily labor	94 (16.5)
	Housewife	124 (21.8)
Partner HIV status	Negative	42 (8.1)
	Positive	181 (35.0)
	Unknown	294 (56.9)
HIV disclosure status	Not disclosed	66 (11.6)
	Disclosed	503 (88.4)

#### Baseline clinical characteristics

Sixty-eight (12%) of the participants were tuberculosis (TB) co-infected and 228 (40%) had at least one OI at entry. Most of the participants 259 (45.5%) were at WHO clinical stage of I and/or II. The other 206 (36%) and 104 (18%) were at WHO stage III and IV respectively. Four hundred twenty-six (75%) were on active working functional status and the other 120 (21.1%) and 23 (4%) were ambulatory and bedridden respectively. The median CD4 count was 266 (IQR = 277). The majority, 392 (69%) of participants were on TDF-3TC-EFV (1e) regimen and in 45 (8%) the original regimen was changed to other regimens due to various reasons mainly adverse drug reactions. Among the total, 159 (28%) had sub-optimal (fair/poor) adherence to ART.

#### Incidence of LTFU

**Fig. 1:**
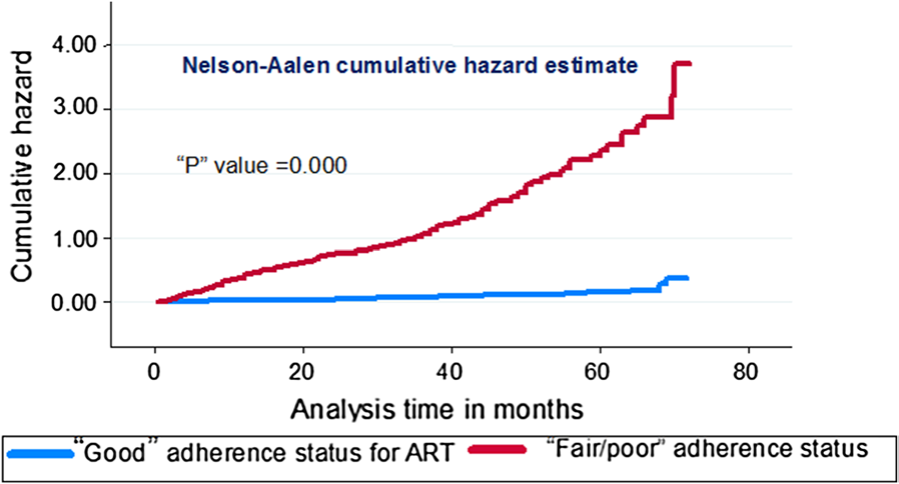
Cumulative hazard estimate of LTFU after initiation of first line ART at University of Gondar comprehensive specialized hospital, Northwest Ethiopia between January 2012 and January 2018 by adherence status

Since the study is a dynamic cohort we have determined the incidence of LTFU by taking the denominator as person year (PY). We had a total of 569 participants who were followed for a total of 1484.11 PY. The overall incidence rate of LTFU was 12.26 per 100 person years (95% CI (10.61–14.18)). The cumulative hazard estimate of LTFU has shown difference among different levels of adherence. The cumulative hazard of LTFU among participants with sub-optimal adherence status (fair/poor) was higher than participants with good adherence status of ART (Fig. 1).

#### Factors affecting lost to follow up from ART care

According to the multivariable Cox regression analysis, underweight patients (BMI < 18.5 kg/m^2^) were 1.52 times at higher risk of LTFU (AHR, 1.52, 95% CI 1.01–2.28) as compared with BMI ≥ 18.5 kg/m^2^. Jobless participants were 2.22 times more likely to be LTFU (AHR, 2.22, 95% CI 1.2–4.11). Participants who are substance abusers had 84% increased risk of LTFU as compared to their counter parts (AHR, 1.84 95% CI 1.19–2.86). Participants with sub-optimal adherence status (fair/poor) had 6.33 times higher risk of LTFU (AHR 6.33, 95% CI 3.90–10.26) than participants with good adherence. Participants with no Isoniazid (INH) prophylaxis were 2.47 times at risk of being loss to follow-up (AHR 2.47, 95% CI (1.36–4.48)) than who received INH prophylaxis. Likewise, participants with ambulatory functional status were 1.94 times at higher risk of LTFU (AHR 1.94, 95% CI (1.23–3.06)) than participants with working functional status. Participants who have OIs were 1.74 times (95% CI 1.11–2.72) more likely to be lost to follow up as compared to their counter parts. When compared with participants with baseline CD4 count of less than 200 cells/µL, those who had 201–349 cells/µL were 42% less likely to be LTFU (AHR 0.58, 95% CI (0.38–0.88)) (Table 2).

**Table 2:** Predictors of LTFU among HIV infected patients after ART initiation in University of Gondar comprehensive specialized Hospital between January 2012 and January 2018

Predictors	Censored n = 387	LTFU n = 182	CHR (95.0% CI)	AHR (95.0% CI)
Baseline CD4 cell				
< 200	138	78	1	1
200–349	120	51	0.65 (0.45–0.92)	0.58 (0.38–0.88)*
350–349	64	36	1.14 (0.76–1.69)	1.37 (0.86–2.20)
≥ 500	65	17	0.75 (0.44–1.28)	1.10 (0.58–2.07)
INH prophylaxis				
Yes	143	17	1	1
No	244	165	4.75 (2.88–7.83)	2.47 (1.36–4.48)**
Adherence status to ART				
Good	373	37	1	1
Fair/poor	14	145	13.98 (9.72–0.10)	6.33 (3.90–10.26)***
Baseline functional status				
Working	323	103	1	1
Ambulatory	56	64	3.16 (2.30–4.33)	1.94 (1.23–3.06)**
Bedridden	8	15	2.92 (1.70–5.03)	1.28 (0.46–3.54)
BMI				
≥ 18.5	322	104	1	1
< 18.5	65	78	2.82 (2.10–3.79)	1.52 (1.01–2.28)*
Presence of OIs				
No	279	69	1	1
Yes	108	113	3.04 (2.25–4.12)	1.74 (1.11–2.72)*
Active TB				
No	367	134	1	1
Yes	20	48	2.84 (2.04–3.97)	0.62 (0.39–1.12)
Substance use				
No	375	140	1	1
Yes	12	42	7.12 (4.99–0.16)	1.84 (1.19–2.86)**
Occupations				
Gov’t/NGO employee	84	24	1	1
Self employed	101	36	1.37 (0.82–2.30)	2.08 (0.96–3.79)
Jobless	76	30	1.68 (0.98–2.88)	2.22 (1.20–4.11)*
Daily labor	50	44	2.53 (1.54–4.16)	1.52 (0.84–2.77)
House wife	76	48	1.84 (1.13–3.01)	1.24 (0.68–2.24)
Marital status				
Married	194	68	1	1
Never married	52	31	2.10 (1.37–3.23)	1.73 (0.99–3.02)
Divorced	104	55	1.72 (1.20–2.47)	1.37 (0.88–2.14)
Widowed/separated	37	28	1.59 (1.04–2.44)	1.32 (0.80–2.17)
HIV disclosure status				
Disclosed	363	140	1	1
Not disclosed	24	42	2.78 (1.97–3. 93)	1.19 (0.77–1.85)
Psychiatric illness				
No	374	155	1	1
Yes	13	27	4.52 (3.15–6.48)	0.82 (0.54–1.26)

***P < 0.001, **P < 0.01 *P < 0.05

### Discussion

The overall incidence of lost to follow up from ART care in our study was 12.26 per 100 person years. This finding is in line with other studies conducted in Gambella, Ethiopia [24] and South Ethiopia [25] but it was higher than a study conducted in Axum Ethiopia [26]. The LTFU incidence in our study is lower when compared with several other studies conducted in Kenya [27], South Africa [28], Guinea-Bissau [29], Cameroon [17], India [30], Latin America and Caribbean [31].

In this study underweight patients were found to be at higher risk of LTFU. This is in line with a study conducted in West Africa, Guinea-Bissau [29] The possible reason as stated by a qualitative study could be many participants who cannot eat regularly, malnourished, drop out of care because of believing that “*medication without proper food is ineffective or even harmful*” [18].

Jobless patients have an increased risk of being LTFU when compared with government/NGO employees. This could be due to lack of money for transport fee, food, etc. which may discourage regular follow-up of ART. Joblessness or unemployment may also represent a marker of more advanced illness [32].

Patients who are substance abusers are more likely to be LTFU which is consistent with another study conducted in Ethiopia [19]. This is because substance use causes negligence and in general decreases the patients’ tendency of receiving and taking the drugs appropriately [33].

Patients with suboptimal adherence were at an increased risk of being lost to follow up when compared with those with good adherence. This was supported by studies conducted in Nigeria [16] and Vietnam [34]. The possible reason could be patients with sub-optimal adherence may have socio-demographic and clinical problems that affect their adherence initially which further affect retention in care [35].

Patients who do not take INH prophylaxis were found to be at higher risk of LTFU. This was consistent with other studies [20, 21, 25]. This could be due to the direct effect of Isoniazid in preventing active tuberculosis, which in turn improves the quality of life of patients which leads to a longer stay in the treatment.

This study showed ambulatory patients to be at an increased risk of being LTFU as compared with working participants. This finding is consistent with study done in Nigeria [36] but another study showed a contrasting result [21]. The possible reason that ambulatory patients are more likely to be LTFU could be due to the social, economical, and financial influences that are caused by their inability to work. Thus may affect their stay in treatment.

Patients who had different opportunistic infections were at higher risk of being LTFU. This result is in line with other studies conducted in Uganda [37] and Togo [15]. This may be because of the pill burden, adverse drug toxicities, and interactions among OI treatment and ART, which demands a high commitment to follow all the medications.

When compared with patients with baseline CD4 count of less than 200 cells/µL, those who had 201–349 cells/µL were 42% less likely to be LTFU. This finding was consistent with other several studies [21, 38, 39]. This might be the fact that an increment in CD4 count improves the immunity of patients and their wellbeing in general which will help them stay on treatment.

## Conclusions

Lost to follow up in the study area was low. Factors that tend to deteriorate the life of patients like malnutrition, OIs, CD4 count below 200 cells/µL, and having suboptimal adherence to ART, ambulatory functional status, being substance abuser and not receiving Isoniazid prophylaxis were significantly associated with LTFU. Therefore clinicians shall consider the identified risk factors while giving ART service and counseling to decrease LTFU.

## Limitations

Since we used secondary data we were not able to include some important variables like distance from the health facility that may affect LTFU.

## Abbreviations

ADL: activity of daily living
AHR: adjusted hazard ratio
AIDS: acquired immunodeficiency syndrome
ART: anti-retroviral therapy
BMI: body mass index
CI: confidence interval
HAART: highly active antiretroviral therapy
HIV: human immunodeficiency virus
INH: isoniazid prophylaxis
IQR: inter-quartile range
LTFU: lost to follow up
NGO: Non-governmental Organization
OIs: opportunistic infections
PLHIV: people living with human immunodeficiency virus
PY: person year of observation
USAID: United States Agency for International Development
WHO: World Health Organization
